# Ultracompact topological photonic switch based on valley-vortex-enhanced high-efficiency phase shift

**DOI:** 10.1038/s41377-022-00993-4

**Published:** 2022-10-10

**Authors:** Hongwei Wang, Guojing Tang, Yu He, Zhen Wang, Xingfeng Li, Lu Sun, Yong Zhang, Luqi Yuan, Jianwen Dong, Yikai Su

**Affiliations:** 1 State Key Laboratory of Advanced Optical Communication Systems and Networks, Department of Electronic Engineering, Shanghai Jiao Tong University, 200240 Shanghai, China; 2 State Key Laboratory of Optoelectronic Materials and Technologies & School of Physics, Sun Yat-sen University, 510275 Guangzhou, China; 3 School of Physics and Astronomy, Shanghai Jiao Tong University, 200240 Shanghai, China

**Keywords:** Photonic crystals, Integrated optics

## Abstract

Topologically protected edge states based on valley photonic crystals (VPCs) have been widely studied, from theoretical verification to technical applications. However, research on integrated tuneable topological devices is still lacking. Here, we study the phase-shifting theory of topological edge modes based on a VPC structure. Benefiting from the phase vortex formed by the VPC structure, the optical path of the topological edge mode in the propagation direction is approximately two-fold that of the conventional optical mode in a strip waveguide. In experiments, we show a 1.57-fold improvement in π-phase tuning efficiency. By leveraging the high-efficiency phase-shifting properties and the sharp-turn features of the topological waveguide, we demonstrate an ultracompact 1 × 2 thermo-optic topological switch (TOTS) operating at telecommunication wavelengths. A switching power of 18.2 mW is needed with an ultracompact device footprint of 25.66 × 28.3 μm in the wavelength range of 1530–1582 nm. To the best of our knowledge, this topological photonic switch is the smallest switch of any dielectric or semiconductor 1 × 2/2 × 2 broadband optical switches, including thermo-optic and electro-optic switches. In addition, a high-speed transmission experiment employing the proposed TOTS is carried out to demonstrate the robust transmission of high-speed data. Our work reveals the phase-shifting mechanism of valley edge modes, which may enable diverse topological functional devices in many fields, such as optical communications, nanophotonics, and quantum information processing.

## Abstract (web summary)

An ultracompact 1 × 2 thermo-optic topological switch is demonstrated by leveraging the high-efficiency phase-shifting property and the sharp-turn feature of the topological waveguides.

## Graphical abstract

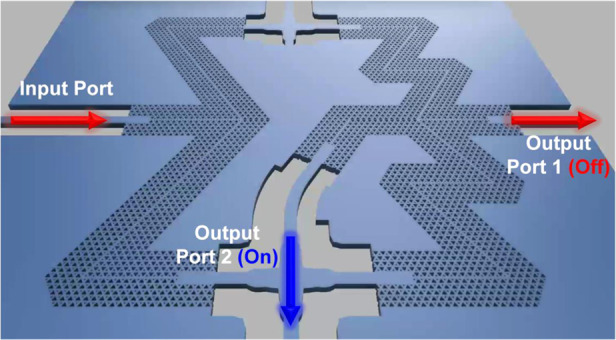

## Introduction

Topological insulators are materials that insulate the interior but support conduction along their edges or surfaces^1,2^. As an important counterpart, topological photonic systems^3–16^ operating in optical frequencies have been extensively studied; these systems are promising for overcoming challenges resulting from fabrication errors where robust transport of light has been widely verified^6,11,15–29^. Although there are many different platforms for photonic topological insulators (PTIs)^4,5^, PTIs with all-dielectric designs have unique advantages due to their compact footprints and low-loss optical transmission^11,15–29^. In particular, valley photonic crystals (VPCs) are one of the most important candidates for future applications in functional devices^20–24^ and have been the subject of substantial research efforts^25–33^. Various topological devices based on valley modes have been proposed, such as optical delay lines^13^, topological lasers^25^, topological photonic routers^27^ and power splitters^31^.

On the other hand, for functional devices for modulation and switching applications, the tunability of the topological optical circuitry is essential. Several tuneable topological devices, such as optically tuneable photonic crystals^29^ and computer-controlled motorized topological insulators^34^, have been proposed. However, it is still challenging to realize an on-chip integrated tuneable device with a compact footprint and a low loss. In conventional integrated tuneable approaches, phase shifters based on thermo-optic (TO) tuning or electro-optic (EO) tuning processes are the key components in on-chip optical circuits^35–37^. However, to the best of our knowledge, the phase-shifting mechanism of topological edge modes has not yet been studied.

Here, we study the phase-shifting mechanism of topological edge modes and propose an ultracompact 1 × 2 thermo-optic topological switch (TOTS) based on VPCs operating at telecommunication wavelengths. We find that the optical path of the topological edge mode in the propagation direction is approximately two-fold that of the conventional optical mode in a strip waveguide with the exploitation of the phase vortex formed by the VPC structure. With an experimental study, we observe that the *π*-phase-shifting efficiency of the topological phase shifter is 1.57 times that of a conventional waveguide-based phase shifter. The high phase-shifting efficiency of the topological waveguide can benefit the thermo-optic switch through a reduced heating length. We, therefore, propose an ultracompact 1 × 2 TOTS that uses a topological Mach–Zehnder interferometer (TMZI) constructed of two 50:50 topological power splitters in VPCs. In the experiment, a switching power of 18.2 mW is required with a device footprint of 25.66 × 28.3 μm in the wavelength range of 1530–1582 nm. To the best of our knowledge, this topological photonic switch is the smallest switch of any dielectric or semiconductor 1 × 2/2 × 2 broadband optical switch due to the high tuning efficiency and the sharp-turn features of the topological waveguide. Furthermore, a high-speed data transmission experiment is carried out to study the switching performance of the proposed TOTS. To the best of our knowledge, this is the first bit error ratio test to demonstrate the robust transmission of high-speed data in a tuneable topological device. The device exhibits a low power penalty of less than 0.5 dB at a 132-Gb/s raw data rate with a four-level pulse amplitude modulation (PAM-4) format. The proposed switch based on a topological phase shifter shows excellent switching performance, which may open routes for the practical applications of topological devices in optical communications, photonic integrated circuits and quantum information processing.

## Results

### Design of VPCs

**Fig. 1:**
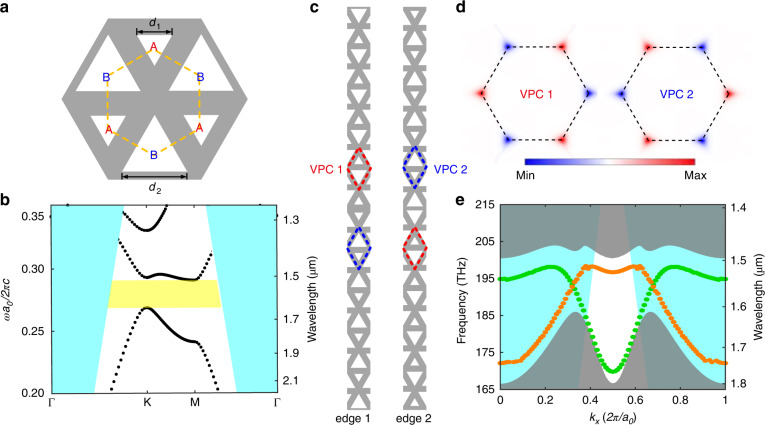
Topological VPC and its bulk band diagram. **a** The dashed hexagon in the schematic presents the unit cell of the VPC slab (*d*_1_ = 122 nm, *d*_2_ = 295 nm). The lattice constant *a*_0_ = 433 nm. **b** Bulk band for both VPC1 and VPC2, where the TE-like polarization bandgap lies between 1500 and 1650 nm. In the first Brillouin zone, Γ, K and M denote the high-symmetry points. **c** Structures for edge 1 and edge 2 in one period along the x-direction, with grey and white regions representing silicon and silica, respectively. The unit cells of VPC1 and VPC2 are outlined with red dashed lines and blue dashed lines, respectively. **d** Berry curvature distribution of the TE_1_ band for VPC1 (left) and VPC2 (right). **e** Edge dispersion of the two edge states supported by edge 1 (green) and edge 2 (orange). The projected bulk band is grey colour. In **b** and **e**, the region above the light cone is cyan colour

In this work, our design consists of a PhC slab based on a silicon-on-insulator wafer with a 220 nm-thick silicon layer. The valley photonic structures comprise two kinds of triangular air holes of different side lengths arranged in a honeycomb lattice, as shown in Fig. 1a. In the presence of inversion symmetry (*d*_1_ = *d*_2_ = 216 nm), the Dirac point appears at approximately *λ* = 1550 nm at the Brillouin zone corners (K and K′) in the band structure. The lattice constant is *a*_0_ = 433 nm. The inversion symmetry of the VPCs can be broken by assigning mismatched wall-length parameters. We define these VPCs as VPC1 (*d*_1_ = 122 nm and *d*_2_ = 295 nm) and VPC2 (*d*_1_ = 295 nm and *d*_2_ = 122 nm), respectively. A bandgap opens between 1500 and 1650 nm, as shown in Fig. 1b. The bulk topology of the transverse-electric (TE)-like band is exhibited by the Berry curvature of the first TE-like band. This special design opens a TE-like polarization bandgap between 1500 and 1650 nm.

In theory, we use an effective Hamiltonian to describe the VPC structure. Based on the plane wave expansion (PWE) method, the effective Hamiltonian with the *k·p* approximation around the K point can be expressed as^27,30^:

1 $$\hat H = v_D\left( {\hat \sigma _x\delta k_x + \hat \sigma _y\delta k_y} \right) + \lambda _{\varepsilon _z}^P\hat \sigma _z$$

where $\hat \sigma _{x,y,z}$ denotes the Pauli matrices, $v_D = \frac{K}{{2\omega _0\mu _0\varepsilon _0}}\left[ {\beta _0 - \frac{1}{2}\left( {\beta _1 + \beta _2} \right)} \right]$is the group velocity, $\lambda _{\varepsilon _z}^P = - \frac{{i\sqrt 3 K^2}}{{8\mu _0\varepsilon _0\omega _0\left( K \right)}}\left( {\beta _1 - \beta _2} \right)$ is the effective mass of Dirac particles, $\omega _0 = \frac{K}{{\sqrt {\mu _0\varepsilon _0} }}\left[ {\beta _0 + \frac{1}{4}\left( {\beta _1 + \beta _2} \right)} \right]^{1/2}$ is the eigenvalue of $\hat H_0$ ($\lambda _{\varepsilon _z}^P = 0$), $\beta _0$, $\beta _1$, and $\beta _2$ are the Fourier expansion coefficients of the constitutive parameters and $K = \frac{{4\pi }}{{3a_0}}$. Then, the valley-dependent topological index around different valleys can be denoted by the Berry curvature, as shown in Fig. 1d. We find that the valley Chern numbers of VPC1 and VPC2 have opposite signs; i.e. *C*_*v1*_ < 0 for VPC1, and *C*_*v2*_ > 0 for VPC2. With the increase of the difference between two triangular holes in size, the profiles of Berry curvature with opposite signs gradually overlap and finally vanish, which results in the deviation of the valley Chern numbers from half integers^27^.

For the valley Hall effect, edge states exist at the interface between the structures with two different ‘polarities’, which in our case are determined by the orientation of the large triangle pointing up (Δ) or down (∇). In the photonic crystal structure of the upper half of edge 1 (edge 2), the large triangles point upwards (downwards), while in the photonic crystal structure of the lower half, the large triangles point downwards (upwards). The differences between the valley indices across interfaces are given by $\Delta C_{{\mathrm{edge}\,{1}}}^K \,>\, 0$, $\Delta C_{{\mathrm{edge}\,{1}}}^{K\prime } \,<\, 0$ for edge 1 and $\Delta C_{{\mathrm{edge}\,{2}}}^K \,<\, 0$, $\Delta C_{{\mathrm{edge}\,{2}}}^{K\prime } \,>\, 0$ for edge 2 and determine the propagation direction of edge states around each valley. Figure 1e shows the dispersion of edge states localized at edge 1 (green curve) and edge 2 (orange curve), named edge state 1 and edge state 2, respectively. The group velocities of the two edge states are almost constant around the valleys, so the effects of the group-velocity dispersion can be ignored for these states. The difference between the relative positions of valleys and light lines in Fig. 1b, e comes from band folding (for details, see Supplementary Note 1).

Furthermore, to quantitatively prove the valley Hall topological protection for these edge states, we study the propagation and reflection of light along an interface with sharp turns (for details, see Supplementary Note 2). In contrast to the PhC waveguide, the back-propagation energy in the topological waveguide before and after the sharp turns are nearly identical. The experimental results prove that the propagation of light along the topological interface based on VPCs is topologically protected.

### Temperature-dependent dispersion relations of the valley edge mode

**Fig. 2:**
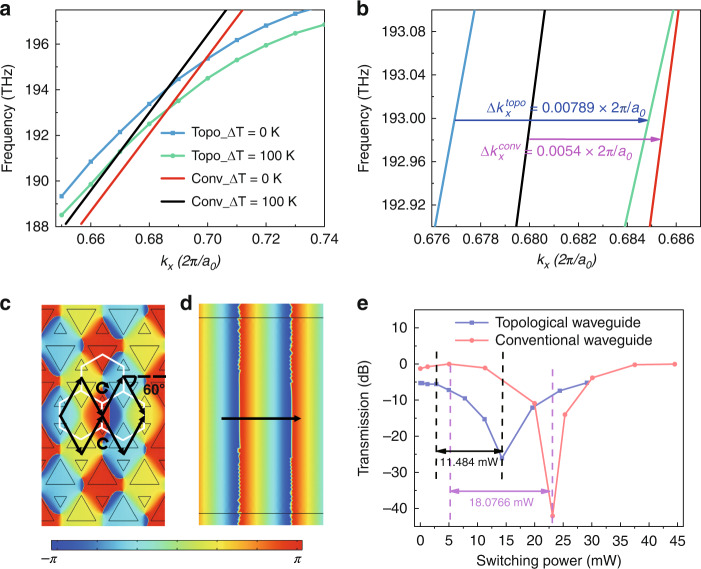
Principle and characterization of the topological phase shifter. **a** Temperature-dependent dispersion relations of the conventional mode and topological mode localized at edge 1. **b** Phase change of the conventional mode and topological mode with a temperature change of 100 K at 193 THz (*λ* = 1550 nm). **c**, **d** The *Hz* phase of the topological mode in edge 1 and the TE_0_ mode in the conventional waveguide. The black arrow represents the direction of light propagation. **e** Switching characteristics of the topological waveguide and the conventional waveguide at the wavelength *λ* = 1550 nm

We analyse the dispersion relation of the edge state based on the VPC using the PWE method. The Dirac equation of edge state 1 can be written as:

2 $$\left[ { - {{{\mathrm{i}}}}\hbar v_D\left( {\sigma _x\partial _x + \sigma _y\partial _y} \right) + \gamma \sigma _z} \right]H_s = \hbar \Omega ( {\delta \mathop{k}\limits^{\rightharpoonup} } )H_s$$

where $\Omega ( {\delta \mathop{k}\limits^{\rightharpoonup} } ) = \omega ( {\delta \mathop{k}\limits^{\rightharpoonup} } ) - \omega _0( K)$ and $$\gamma \left( y \right) = \left\{ {\begin{array}{*{20}{c}} {\lambda _{\varepsilon z}^P,\quad y \,>\, 0} \\ {0,\quad y = 0} \\ { - \lambda _{\varepsilon z}^P,\quad y \,<\, 0} \end{array}} \right.$$. According to the Jackiw–Rebbi theory, we can obtain the dispersion relation of edge state 1, which is $\omega ( {\delta \mathop{k}\limits^{\rightharpoonup} }) = \frac{{KcC_{s1}^{\frac{1}{2}}}}{{n_{{\mathrm{eff}}}}} + \frac{{cC_{s2}}}{{2n_{{\mathrm{eff}}}C_{s1}^{\frac{1}{2}}}}\delta k$ around the K point (for details, see Supplementary Note 1). The dispersion is related to the effective refractive index $n_{{\mathrm{eff}}}$ of the silicon-on-insulator wafer, which can be tuned by changing the doped carrier concentration or temperature. As an example, at a certain frequency, the differential value of the phase with temperature can be expressed as:

3 $$\Delta \phi _{{{{\mathrm{topo}}}}} = - \frac{{2KC_{s1}}}{{n_{{\mathrm{eff}}}C_{s2}}}\frac{{dn_{{\mathrm{eff}}}\left( T \right)}}{{dT}}l_{{{{\mathrm{topo}}}}}\Delta T_1$$

where $C_{s1} \approx 1.71$, $C_{s2} \approx 2.01$, $n_{{\mathrm{eff}}} \approx 2.8323$ and $l_{{{{\mathrm{topo}}}}}$ is the heating length. For the conventional waveguide, the temperature-dependent phase shift is $\Delta \phi _{{{{\mathrm{trad}}}}} = \frac{{2\pi }}{\lambda }\frac{{dn_{{\mathrm{eff}}}\left( T \right)}}{{dT}}l_{{{{\mathrm{trad}}}}}\Delta T_2$. This waveguide requires a higher heating temperature ($\Delta T_1 = 1.44\Delta T_2$) to achieve the same π-phase shifting as that of a topological waveguide with the same heating length ($l_{{{{\mathrm{trad}}}}} = l_{{{{\mathrm{topo}}}}}$). As shown in Fig. 2a, to comprehensively study the property of the phase in valley-dependent edge states, we simulate the energy band of the valley edge states and obtain the temperature-dependent dispersion relations of a conventional mode and a topological mode localized at edge 1. Considering the dispersion, the group-velocity *v*_*g*_ (the slope in the dispersion of the topological modes) is actually the slope of the bulk band around the Dirac point. The Dirac point is located at the boundary of the Brillouin zone (K/K′ point). The photonic band usually has a small slope near the boundary of the Brillouin zone. Therefore, topological edge modes tend to have lower group velocities than conventional modes. The simulation results in Fig. 2b show that the phase change of the valley edge mode ($\Delta k_x^{{\mathrm{topo}}} = 0.00789 \times 2\pi /a_0$) is 1.46 times that of the conventional waveguide ($\Delta k_x^{{\mathrm{trad}}} = 0.0054 \times 2\pi /a_0$) with the same refractive index variation of silicon. In addition, the topological mode at edge 1 has a phase shift efficiency of 1.547 times that of the conventional PhC waveguide (W1 waveguide) (for details, see Supplementary Note 1). As illustrated in Reference ^31^, edge state 1 in Fig. 1c can be expressed by two valley-vortex states $\left| {{\mathrm{LCP}}} \right\rangle$ and $\left| {{\mathrm{RCP}}} \right\rangle$as $\left| {\psi _{{\mathrm{edge}}\,1}^K} \right\rangle = \frac{1}{{\sqrt 2 }}\left( {\left| {{\mathrm{LCP}}} \right\rangle + \left| {{\mathrm{RCP}}} \right\rangle } \right)e^{ - \mathop{\alpha }\limits^{\rightharpoonup} \left( {\mathop{r}\limits^{\rightharpoonup} } \right) \cdot \mathop{r}\limits^{\rightharpoonup} }$. Benefiting from the phase vortex produced by the VPC structure^27,28^, the length of the optical path of the topological mode is close to two-fold that of the classical mode at *λ* = 1550 nm, as illustrated in Fig. 2c, d.

Furthermore, we employ a conventional Mach–Zehnder interferometer structure to calculate the π-phase-shifting power for these two types of waveguides (for details, see Supplementary Note 3). The experimental results agree well with the theoretical and simulation results. As shown in Fig. 2e, the *π*-phase-shifting powers of the topological phase shifter and the conventional waveguide-based phase shifter are 11.484 and 18.07 mW with the same heating length, respectively. Therefore, the experimental results show that topological edge modes are promising candidates for realizing low power and compact modulators or switches in integrated optical networks.

#### Topological switch

**Fig. 3:**
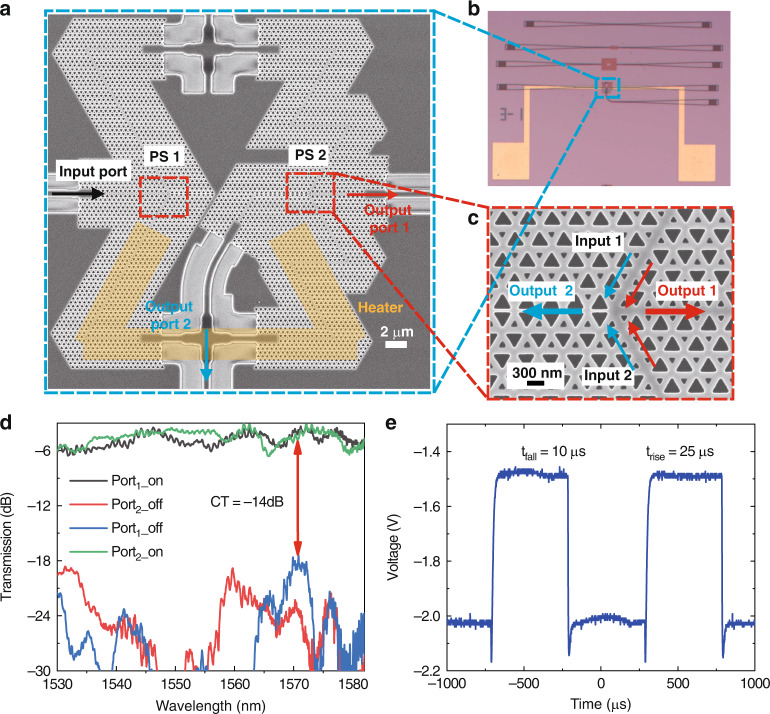
A topological switch with topologically protected valley edge modes. **a** Scanning electron microscope (SEM) image and **b** Microscope image of the topological switch. The orange region in the **a** represents the “Heater”. **c** SEM image of PS2. **d** Normalized transmission spectra of the fabricated topological switch. **e** Temporal responses of the switch

Based on the topological phase shifter, we propose an ultracompact 1 × 2 TOTS, as shown in Fig. 3a. The TOTS consists of two power splitters (PS), two crossing structures (see Supplementary Note 7) and two symmetrical TMZI arms. The footprint of the TOTS is 25.66 × 28.3 μm. Benefiting from the high phase-shifting efficiency and sharp-turn transport of the topological waveguides, the *π* phase shift can be realized in a smaller footprint based on the VPC structure. Details of the fabrication process and the experimental setup can be found in the Materials and methods section^38,39^. The output of the light can be tuned by changing the phase of the input signal before power splitter 2 (for details, see Supplementary Note 5). A microscope image of the TOTS is shown in Fig. 3b. The heating length *L* of the straight section in the TMZI arms is 48 μm. When the TOTS is turned “off”, light passes through Port 1 with a low loss (i.e. no extra phase change is introduced for the MZI arms). When an extra phase change of π is added to one of the TMZI arms (the TOTS is turned “on”), destructive interference occurs at PS 2, as shown in Fig. 3c, and light is output from Port 2 with a low loss.

Figure 3d shows the measured transmission of the TOTS with operating wavelengths in the range of 1530–1582 nm. When the tuning power of the heater is increased to 18.2 mW, the output port of the device is switched from Port 1 to Port 2. In the wavelength range of 1530–1582 nm, the crosstalk at Port 1 (off-state) is −14 dB, and the insertion loss at Port 2 (on-state) is 6.2 dB. The measured transmission responses are normalized to those of the straight topological waveguide fabricated on the same chip, as shown in Fig. 3b. The operating bandwidth of the TOTS is related to the dispersion relation of the topological waveguides (see Supplementary Note 6). In addition, an on-off switching test is performed to measure the response time of the TOTS. We apply a 10 kHz square-wave electrical signal to the heater, as shown in Fig. 3e. Then, an output signal is obtained at Port 1. This signal shows that Port 1 has a rise time constant of 25 µs and a fall time constant of 10 µs.

### High-speed robust transmission

**Fig. 4:**
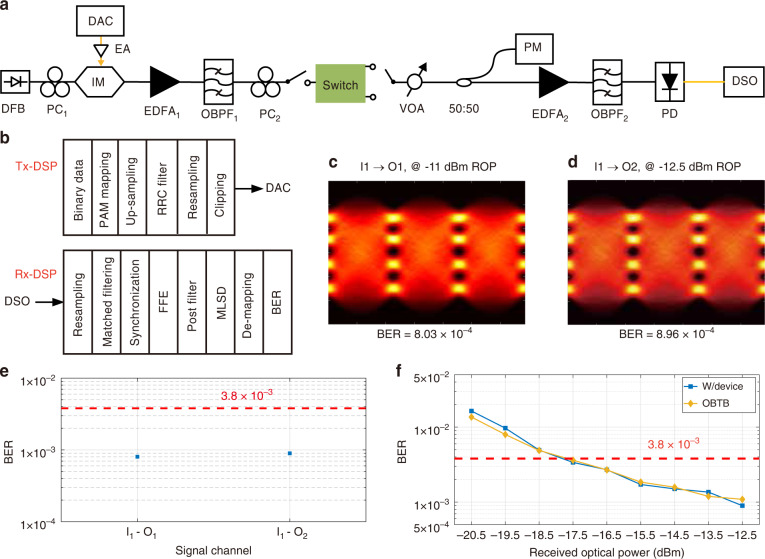
High-speed data transmission experiment based on the proposed topological switch. **a** Experimental setup and **b** DSP flow charts for the high data rate switching of the device. PM power metre. **c, d** Eye diagrams for the two switching configurations of the switch. **e** BERs of two switching configurations. **f** BER versus received optical power (ROP)

To further explore the applications of tuneable topological devices in optical transmission systems, we perform a high-speed data transmission experiment using the proposed topological switch for on-chip communications. Here, we provide the bit error ratio (BER) measurement through the TOTS for both output ports with 132 Gb/s four-level pulse amplitude modulation (PAM-4) data. To the best of our knowledge, this is the first bit error ratio test that proves the robust transmission of high-speed data in a tuneable topological device. Figure 4a, b shows the experimental setup and the transceiver digital signal processing (DSP) flow charts. The eye diagrams of the recovered PAM-4 signals at different switching configurations are provided in Fig. 4c, d. Furthermore, we evaluate the BER performance of the 66-GBaud PAM-4 signal in the presence of optical switching, as illustrated in Fig. 4e. The 7% hard-decision forward error correction threshold of 3.8 × 10^−3^ is achieved for all the switching configurations. We also measure the BER for the worst switching state (Output Port 2), as shown in Fig. 4f. Compared to the optical back-to-back (OBTB) sensitivity of −18 dBm, there is no penalty for the switching signal, indicating that the bandwidth of the proposed switch is wide enough for high-capacity switching at a 132 Gb/s rate. The high-speed transmission experiment demonstrates that the proposed TOTS can be used as a basic functional device for on-chip communication.

Table 1 compares our device with other reported 1 × 2/2 × 2 switches based on different mechanisms. Our TOTS exhibits the smallest footprint of dielectric or semiconductor 1 × 2/2 × 2 broadband switches to the best of our knowledge. A plasmonic switch with metal waveguides is smaller at the cost of a high driving voltage and limited propagation length^40^. There are two reasons for the compact size of the proposed TOTS: one is the reduced heating length resulting from the high phase-shifting efficiency, and the other is the sharp turns of the topological waveguides, which more efficiently use the footprint. Our work also shows comparable performance in terms of the largest working bandwidth (>42 nm), large extinction ratio in output ports (>14 dB), high data rate (132 Gb/s) and fast switching time (25 μs/10 μs). The long switching time of the proposed switch is attributed to the slow thermo-optical response of silicon. Based on the Pockels effect of LiNbO_3_^40–43^, VPC-based topological waveguides in LiNbO_3_ photonic crystal plates can achieve high-speed topological switching. However, due to the low refractive index of LiNbO_3_, the topological devices based on the LiNbO_3_ platform have larger footprints and higher out-of-plane radiations than silicon-based devices. Therefore, LiNbO_3_-based topological switches exhibit larger footprints and higher insertion losses than silicon-based topological switches, while the EO performance is better.

**Table 1:** Comparison of various on-chip 1 × 2/2 × 2 broadband switches

Type	Structure	Material(s)	Mechanism	Footprint (μm^2^)	Switching power (mW)	IL (dB)	ER (dB)	Bandwidth (nm)	Data rate (Gb/s)	Modulation format	Switching time (μs)
^34^	Topological switch	Copper	Motorized	~35 cm × 40 cm	—	—	—	—	—	—	4 s
^35^	MZI	Silicon	TO	>200 × 30	—	1	20	148	—	—	—
^44^	MZI	Silicon	TO	~160 × 20	28	1.3	15	60	—	—	—
^45^	DC	Silicon	MEMS	∼110 × 110	24 V	1	15	120	—	—	0.91
^46^	MZI	Sb_2_Se_3_	PCMs	>100 × 100	18.6	0.36	6.5 /15.0	15	—	—	0.1–1 ms/800 ns
^36^	MZI	Silicon	EO	>400 × 50	2.475	2	—	—	—	—	24 ns
^37^	MZMs	Si/LN	EO	>5000 × 50	5.1 V	2.5	40	40	100	OOK	—
^40^	DC	LN/Au	EO	25 × 10	50 V	5.5	10	310	—	—	~fs
Our work	Topological MZI	Silicon	TO	25.7 × 28.3	18.2	6.2	14	~42	132	PAM-4	25/10

*PCMs* phase change materials, *ER* extinction ratio, *IL* insertion loss, *MZI* Mach–Zehnder interferometer, *MEMS* microelectromechanical systems, *DC* directional coupler, *EO* electro-optic effect, *TO* thermo-optic effect, *LN* LiNbO3, *MZM* Mach–Zehnder modulators, *Au* gold

## Discussion

In conclusion, we studied the phase-shifting theory of topological edge modes based on a VPC structure and proposed an on-chip ultracompact 1 × 2 thermo-optic topological switch (TOTS). The length of the optical path of the topological edge mode is close to two-fold that of the conventional mode at the same propagation length due to the phase vortex formed by the VPC structure. The experimental results show that the π-phase switching efficiency of TMZI is 1.57-fold that of the conventional MZI. Benefiting from the high phase-shifting efficiency and robust transport of the topological waveguides, we design an ultracompact 1 × 2 topological thermo-optical switch based on high phase-shifting efficiency and robust transport of the topological edge states. Furthermore, we characterize the properties of the fabricated TOTS by testing the transmission of the device. In the experiment, a switching power of 18.2 mW is required with a device footprint of 25.66 × 28.3 μm. To the best of our knowledge, this TOTS is the smallest switch of dielectric or semiconductor 1 × 2/2 × 2 broadband optical switches. The results show a low power penalty of less than 0.5 dB at a 132 Gb/s raw data rate with a PAM-4 formats. This work realizes on-chip tuneable topological devices in photonic topological insulators, which may enable diverse topological functional devices in many fields, such as optical communications, nanophotonics, and quantum information processing.

## Materials and methods

### Sample fabrication

We fabricated the samples to perform the transmission measurements. The proposed topological devices were fabricated on a silicon-on-insulator (SOI) wafer with a 220 nm-thick top silicon layer and a 3 μm-thick buried silica layer. The silicon waveguides and VPC structures were etched to a depth of 220 nm, and grating couplers were shallowly etched to a depth of 70 nm. The devices were fabricated by using E-beam lithography technology (Vistec EBPG 5200^+^) and an inductively coupled plasma etching process (SPTS DRIE-I). After the deposition of the silica upper-cladding layer, 350 nm-thick and 2.4 μm-wide titanium heaters and gold contact pads were fabricated on the waveguides by a lift-off process using an e-beam evaporator. A SiO_2_ layer is needed between the silicon waveguide and the metallic microheater to avoid optical absorption induced by metals. The details of the fabrication processes of the structures proposed in this work can be found in refs. ^38,39^.

### Optical characterization

A tuneable continuous-wave laser (Santec TSL770) and an optical power metre (Keysight N7744A) were used to characterize the topological devices. The grating couplers were used to couple the light into/out of the chip. The period and duty cycle of the grating coupler were 630 nm and 50%, respectively. The etching depth was 70 nm. The coupling loss was 8 dB/port at the central wavelengths of the grating coupler. The optical signal was coupled to the silicon chip through the input grating coupler (see Supplementary Note 8). An intermediate waveguide was introduced to connect the conventional waveguide and the topological structure to reduce the mode-wave-vector mismatch and suppress scattering to the air. The design of the intermediate waveguide is discussed in ref. ^31^. By using a voltage-current source-metre (Keithley 2400), we measured the thermal tuning efficiency of the TOTS by applying different voltages to the gold contact pads.

### Numerical simulation

We studied the bulk topology of the transverse-electric (TE)-like band and the band diagrams of the two edge states, as shown in Figs. 1b, e and 2a, by using the MIT Photonic Bands (MPB) package. The refractive index of silicon was taken as *n*_*Si*_ = 3.48. The plane wave expansion method was used to calculate the dispersion relation of topological modes based on an effective Dirac Hamiltonian around the K point, as shown in Supplementary Note 1.

### Experimental setup of high-speed transmission

The experimental setup of high-speed transmission is shown in Fig. 4a, b. A PAM-4 data stream is upsampled twice at the transmitter before being processed by a root-raised cosine filter with a roll-off factor of 0.01. After resampling, the PAM-4 signal is sent to the 100 GSa/s detail-to-analogue converter (DAC) (MICRAM DAC 1002). An electrical amplifier amplifies the DAC’s output signal, which then drives a 25 GHz IM biased at the quadrature point of its transmission curve. The IM is injected with continuous-wave light from a distributed feedback laser. An erbium-doped fibre amplifier (EDFA) boosts the optical PAM-4 signal after electrical-to-optical conversion, followed by an optical bandpass filter (OBPF) to minimize the increased spontaneous emission noise. After the OBPF, the polarization state of the PAM signal is adjusted with a polarization controller before entering the silicon chip. A variable optical attenuator is inserted at the output port of the topological device to adjust the received optical power. The receiver contains an EDFA for preamplification, an OBPF, a 43 GHz photodetector (PD), and an 80 GSa/s digital storage oscilloscope (LeCroy 36Zi-A). In the receiver DSP, the signal is first resampled to a sampling rate of two samples per symbol. We apply an 81-tap linear feedforward equalizer (FFE) after synchronization and matched filtering. The least mean square (LMS) technique is used to derive the equalization coefficients from the training sequence. To minimize the influence of the noise enhancement effect of the FFE, a 2-tap poster filter and the maximum-likelihood sequence decision are implemented. Finally, the PAM-4 demapping and BER calculation are performed.

## Supplementary information

Supplementary Information for Ultracompact topological photonic switch based on valley-vortex-enhanced high-efficiency phase shift
