# Supplementary material for: Ultracompact topological photonic switch based on valley-vortex-enhanced high-efficiency phase shift
Source: Light Sci Appl. 2022 Oct 10;11:292. doi: 10.1038/s41377-022-00993-4 (PMC9551041; doi:10.1038/s41377-022-00993-4)
Supplement: Supplementary file 1 — Supplementary Information for Ultracompact topological photonic switch based on valley-vortex-enhanced high-efficiency phase shift [file 41377_2022_993_MOESM1_ESM.pdf]

Supplementary Information for

Ultracompact topological photonic switch based on valley-vortex-enhanced high-efficiency phase shift

Hongwei Wang[1†], Guojing Tang[2†], Yu He[1], Zhen Wang[1], Xingfeng Li[1], Lu Sun[1], Yong Zhang[1], Luqi Yuan[3*], Jianwen Dong[2*] and Yikai Su[1*]

1. State Key Laboratory of Advanced Optical Communication Systems and Networks, Department of Electronic Engineering, Shanghai Jiao Tong University, Shanghai 200240, China

2. State Key Laboratory of Optoelectronic Materials and Technologies & School of Physics, Sun Yat-sen University, Guangzhou 510275, China

3. School of Physics and Astronomy, Shanghai Jiao Tong University, Shanghai 200240, China

* Correspondence: Yikai Su (yikaisu@sjtu.edu.cn)

Department of Electronic Engineering

Shanghai Jiao Tong University

Shanghai 200240, China

Email: yikaisu@sjtu.edu.cn

Phone: +86 021-34204356

* Correspondence: Jianwen Dong (dongjwen@mail.sysu.edu.cn)

State Key Laboratory of Optoelectronic Materials and Technologies & School of Physics

Sun Yat-sen University

Guangzhou 510275, China

Email: dongjwen@mail.sysu.edu.cn

Phone: +86 020-84111469

* Correspondence: Luqi Yuan (yuanluqi@sjtu.edu.cn)
School of Physics and Astronomy,
Shanghai Jiao Tong University,
Shanghai 200240, China
Email: yuanluqi@sjtu.edu.cn;
Phone: +86-13817347573

†These authors contributed equally to this work.

Supplementary Note 1: The temperature-dependent dispersion relation of the valley edge state

The dispersion relation of the valley edge state is discussed in this section. The Hamiltonian is first deduced analytically to characterize the band diagram of the valley-Hall topological photonic crystal (PhC) insulator in two time-reversal but inequivalent valleys: K' and K. By using the plane wave expansion (PWE) method[1-3], we consider the TE modes and convert the wave equations for electromagnetic fields into an eigenvalue problem described by a Hamiltonian. The corresponding Maxwell equations can be written as:

$$\partial_y H_z = -i\omega\varepsilon_0\varepsilon_r E_x,\quad \partial_x H_z = i\omega\varepsilon_0\varepsilon_r E_y,\quad \partial_x E_y - \partial_y E_x = i\omega\mu_0 H_z, \tag{1}$$

By expressing E_x and E_y in terms of H_z, we obtain:

$$\omega^2\mu_0\varepsilon_0\varepsilon_z H_z + \partial_x\left(\frac{1}{\varepsilon_r}\partial_x H_z\right) + \partial_y\left(\frac{1}{\varepsilon_r}\partial_y H_z\right) = 0. \tag{2}$$

Using the VPC periodicity and PWE method, the field and constitutive parameters can be expressed as $H_z(\vec{r},\vec{q}) = \sum_{\vec{G}} H_{\vec{G}} e^{i(\vec{q}+\vec{G})\cdot\vec{r}}$, $\frac{1}{\varepsilon_r}(\vec{r}) = \sum_{\vec{G}} \beta_{\vec{G}} e^{i\vec{G}\cdot\vec{r}}$. Vectors $\vec{G} = \vec{G}_i, (i = 0,1,2)$ denote the plane wave basis of the K valley, which is truncated to the first three waves containing the shortest reciprocal vectors: $\vec{G}_0 = [0,0]$, $\vec{G}_1 = [-3K/2, -\sqrt{3}K/2]$, and $\vec{G}_2 = [-3K/2, \sqrt{3}K/2]$. The linear equations for the Fourier components of H_z can be obtained by substituting them into Eq. (2):

$$\omega^2\mu_0\varepsilon_0 H_{\vec{G}} - \sum_{\vec{G}'} \beta_{\vec{G}-\vec{G}'}[(\vec{q}+\vec{G})\cdot(\vec{q}+\vec{G}')]H_{\vec{G}'} = 0\,. \tag{3}$$

Therefore, we investigate the waves propagating with wave vectors $q_x = K + \delta k_x$ and $q_y = \delta k_y$ in the vicinity of the K point, where $K = 4\pi/3a_0$ and δk_x and δk_y are small perturbations with respect to the K point. Then, Eq. 3 can be simplified to:

$$\omega^2\mu_0\varepsilon_0\tilde{H} = \hat{\alpha}_{PW} \odot \hat{\beta}_{PW}\tilde{H} \tag{4}$$

where $\hat{\alpha}_{PW} = K^2\begin{pmatrix} 2 & -1 & -1 \\ -1 & 2 & -1 \\ -1 & -1 & 2 \end{pmatrix} + K\delta k_x \begin{pmatrix} 2 & \frac{1}{2} & \frac{1}{2} \\ \frac{1}{2} & -1 & -1 \\ \frac{1}{2} & -1 & -1 \end{pmatrix} + K\delta k_y \begin{pmatrix} 0 & \frac{\sqrt{3}}{2} & -\frac{\sqrt{3}}{2} \\ \frac{\sqrt{3}}{2} & \sqrt{3} & 0 \\ -\frac{\sqrt{3}}{2} & 0 & -\sqrt{3} \end{pmatrix}$, $\hat{\beta}_{PW} = \begin{pmatrix} \beta_{\vec{G}_0} & \beta_{\vec{G}_0-\vec{G}_1} & \beta_{\vec{G}_0-\vec{G}_2} \\ \beta_{\vec{G}_1} & \beta_{\vec{G}_0} & \beta_{\vec{G}_1-\vec{G}_2} \\ \beta_{\vec{G}_2} & \beta_{\vec{G}_2-\vec{G}_1} & \beta_{\vec{G}_0} \end{pmatrix}$, $\tilde{H} = \left[H_{G_0}; H_{G_1}; H_{G_2}\right]$

and "⊙" is the Hadamard product. With the C_3 symmetry, we have $\beta_{\vec{G}_0-\vec{G}_1} = \beta_{\vec{G}_2}$, $\beta_{\vec{G}_0-\vec{G}_2} = \beta_{\vec{G}_1}$, $\beta_{\vec{G}_1-\vec{G}_2} = \beta_{\vec{G}_2}$, $\beta_{\vec{G}_2-\vec{G}_1} = \beta_{\vec{G}_1}$. Then, $\hat{\beta}_{PW}$ can be simplified as $\hat{\beta}_{PW} = \begin{pmatrix} \beta_{\vec{G}_0} & \beta_{\vec{G}_2} & \beta_{\vec{G}_1} \\ \beta_{\vec{G}_1} & \beta_{\vec{G}_0} & \beta_{\vec{G}_2} \\ \beta_{\vec{G}_2} & \beta_{\vec{G}_1} & \beta_{\vec{G}_0} \end{pmatrix} = \begin{pmatrix} \beta_0 & \beta_2 & \beta_1 \\ \beta_1 & \beta_0 & \beta_2 \\ \beta_2 & \beta_1 & \beta_0 \end{pmatrix}$. Then, we express this matrix in a new basis $\tilde{H}_{spin} = [H_S; H_R; H_L]$ based on a singlet state H_S and two doublet states H_R and H_L. By using the transformation $\hat{U} = \frac{\sqrt{3}}{3}\left[1,1,1;1,\eta^2,\eta;1,\eta,\eta^2\right]$ where $\eta = \exp(i2\pi/3)$, Eq. 4 can be written as:

$$\omega^2\mu_0\varepsilon_0\tilde{H}_{spin} = (K^2\hat{W} + K\delta k_x\hat{X} + K\delta k_y\hat{Y})\tilde{H}_{spin}\,, \tag{5}$$

where

$$\hat{W} = \begin{pmatrix} \beta_0 - \frac{1}{2}(\beta_1+\beta_2) & 0 & 0 \\ 0 & \beta_0 - \frac{1}{2}(\eta\beta_1 + \eta^2\beta_2) & 0 \\ 0 & 0 & \beta_0 - \frac{1}{2}(\eta^2\beta_1 + \eta\beta_2) \end{pmatrix},$$

$$\hat{X} = \begin{pmatrix} 0 & \beta_0 - \frac{1}{2}(\eta^2\beta_1 + \eta\beta_2) & \beta_0 - \frac{1}{2}(\eta\beta_1 + \eta^2\beta_2) \\ \beta_0 - \frac{1}{2}(\eta^2\beta_1 + \eta\beta_2) & 0 & \beta_0 - \frac{1}{2}(\beta_1+\beta_2) \\ \beta_0 - \frac{1}{2}(\eta\beta_1 + \eta^2\beta_2) & \beta_0 - \frac{1}{2}(\beta_1+\beta_2) & 0 \end{pmatrix},$$

$$\hat{Y}=\begin{pmatrix} 0 & -i\left[\beta_0-\frac{1}{2}\left(\eta^2\beta_1+\eta\beta_2\right)\right] & i\left[\beta_0-\frac{1}{2}\left(\eta\beta_1+\eta^2\beta_2\right)\right] \\ i\left[\beta_0-\frac{1}{2}\left(\eta^2\beta_1+\eta\beta_2\right)\right] & 0 & -i\left[\beta_0-\frac{1}{2}\left(\beta_1+\beta_2\right)\right] \\ -i\left[\beta_0-\frac{1}{2}\left(\eta\beta_1+\eta^2\beta_2\right)\right] & i\left[\beta_0-\frac{1}{2}\left(\beta_1+\beta_2\right)\right] & 0 \end{pmatrix}.$$

The eigenfrequency of the singlet state is often far from those of the degenerate doublet states near the K point.[3] Therefore, we can ignore the impact of the singlet state in further considerations when the interactions between doublet states are around the K point. The eigenvalue equation can be simplified as:

$$\begin{aligned}&\left[\omega^2\mu_0\varepsilon_0-K^2\begin{pmatrix}\beta_0-\frac{1}{2}\left(\eta\beta_1+\eta^2\beta_2\right) & 0\\ 0 & \beta_{G0}-\frac{1}{2}\left(\eta^2\beta_1+\eta\beta_2\right)\end{pmatrix}\right]\begin{bmatrix}H_R\\ H_L\end{bmatrix}\\ &=\left[\beta_0-\frac{1}{2}\left(\beta_1+\beta_2\right)\right]K\left(\delta k_x\hat{\sigma}_x+\delta k_y\hat{\sigma}_y\right)\begin{bmatrix}H_R\\ H_L\end{bmatrix}\end{aligned}. \tag{6}$$

The eigenvalue of Eq. 6 is $\omega_0=\frac{K}{\sqrt{\mu_0\varepsilon_0}}\left[\beta_0+\frac{1}{4}(\beta_1+\beta_2)\right]^{1/2}$ when $\beta_1=\beta_2$. To obtain Dirac equations by eliminating the monopolar state, we use $\omega=\omega_0+\Omega(\delta k)$, where $\Omega(\delta\vec{k})$ is a small perturbation. Then, we can obtain that $\omega^2\overset{\Omega\to 0}{\to}\omega_0^2+2\Omega(\delta k)\omega_0$. The effective Hamiltonian can be obtained through the $k{\cdot}p$ approximation[2,3]:

$$[v_D(\hat{\sigma}_x\delta k_x+\hat{\sigma}_y\delta k_y)+\lambda_{\varepsilon_z}^P\hat{\sigma}_z]H_s=\Omega(\delta\vec{k})H_s, \tag{7}$$

where $\hat{\sigma}_x$, $\hat{\sigma}_y$, and $\hat{\sigma}_z$ are Pauli matrices, $v_D=\frac{K}{2\omega_0\mu_0\varepsilon_0}\left[\beta_0-\frac{1}{2}\left(\beta_1+\beta_2\right)\right]$, $\lambda_{\varepsilon_z}^P=-\frac{i\sqrt{3}K^2}{8\mu_0\varepsilon_0\omega_0(K)}(\beta_1-\beta_2)$, and $H_s=\begin{bmatrix}H_R\\ H_L\end{bmatrix}$.

In our design of topological PhCs, β_0, β_1 and β_2 can be expressed as $\beta_i=\frac{1}{S_{cell}}\int\frac{1}{\varepsilon}(\vec{r})e^{-i\vec{G}_i\cdot\vec{r}}dS$. After simple numerical calculations, we can obtain that $\beta_1+\beta_2=\frac{1}{S_{cell}}\int\frac{1}{\varepsilon}(\vec{r})2\cos(\vec{G}_1\cdot\vec{r})dS\approx-0.41\frac{1}{\varepsilon_{Si}}$ and $\beta_0=\frac{1}{S_{cell}}\int\frac{1}{\varepsilon}(\vec{r})dS\approx 1.81\frac{1}{\varepsilon_{Si}}$. We denote $\beta_0+\frac{1}{4}\left(\beta_1+\beta_2\right)=C_{s1}\frac{1}{\varepsilon_{Si}}$ and $\beta_0-\frac{1}{2}\left(\beta_1+\beta_2\right)=C_{s2}\frac{1}{\varepsilon_{Si}}$, where $C_{s1}\approx 1.71$ and $C_{s2}\approx 2.01$. Then, the eigenvalue can be written as $\omega_0(K)=\frac{cKC_{s1}^{\frac{1}{2}}}{n_{eff}}=\frac{4\pi cC_{s1}^{\frac{1}{2}}}{3a_0n_{eff}}$, where n_{eff} is the effective refractive index of silicon and c is the speed of light in vacuum.

In quantum mechanics, the position and momentum coordinates satisfy the following relationships: $\delta k_x=-i\hbar\partial_x$ and $\delta k_y=-i\hbar\partial_y$. By substituting the relation into Eq. 7, we can obtain the Dirac equation written as[4]:

$$[-i\hbar v_D(\sigma_x\partial_x+\sigma_y\partial_y)+\gamma\sigma_z]H_s=\hbar\Omega(\delta\vec{k})H_s, \tag{8}$$

The solution to this equation is the famous Jackiw-Rebbi solution[1]. We can obtain that $\Omega(\delta\vec{k})=\omega(\delta\vec{k})-\omega_0(K)=v_D\delta k$. Thus, the dispersion relation of the valley edge states is:

$$\omega(\delta\vec{k})=\frac{KcC_{s1}^{\frac{1}{2}}}{n_{eff}}+\frac{cC_{s2}}{2n_{eff}C_{s1}^{\frac{1}{2}}}\delta k. \tag{9}$$

As shown in Fig. 1b and Fig. 1e, there is a difference between the relative positions of valleys and light lines, which originates from band folding. The bulk band in Fig. 1b describes the band structure of unit cells (orange dashed line in Fig. 1a) with a periodic hexagonal lattice. The Brillouin zone of the unit cells is a hexagon with a side length of $\frac{4\pi}{3a_0}$. The edge dispersion describes the band structure of the supercells with domain walls (structures in Fig. 1c). The supercell is only periodic in the direction of the domain wall, and the Brillouin zone changes into a 1D segment with a length of $\frac{2\pi}{a_0}$. When the periodic unit cells are transformed into supercells, the Brillouin zone also changes. The K (K') point at $(k_x,k_y)=(\frac{4\pi}{3a_0},0)$ ($(k_x,k_y)=(-\frac{4\pi}{3a_0},0)$) is out of the Brillouin zone of supercells. According to the period value of the photonic band, the regions out of the Brillouin zone need to be folded into the Brillouin zone by translating the integer times the reciprocal lattice vector. The K (K') point is folded to $k_x=-\frac{2\pi}{3a_0}$ ($k_x=\frac{2\pi}{3a_0}$). Therefore, the valley is in the region above the light line after band folding. Similar situations, i.e., a "change" in the position of the valley, can be found in many other previous works on photonic crystal slabs[3,5-7].

The band structure shifts in frequency when the refractive index of silicon is changed:

$$\Delta\omega=\frac{KcC_{s1}^{\frac{1}{2}}}{n_{eff}^{2}}\Delta n_{eff}. \tag{10}$$

Then, the wave vector variation at a fixed frequency and the refractive index variation (Δn_{eff}) satisfy the following relation:

$$\Delta k=\frac{2n_{eff}C_{s1}^{\frac{1}{2}}}{cC_{s2}}\Delta\omega=\frac{2KC_{s1}}{n_{eff}C_{s2}}\Delta n_{eff}. \tag{11}$$

Compared with the change in the wave vector for a conventional waveguide $\Delta k=k_0\Delta n_{eff}$, the edge states localized at the domain wall between VPCs have higher phase-shifting efficiency.

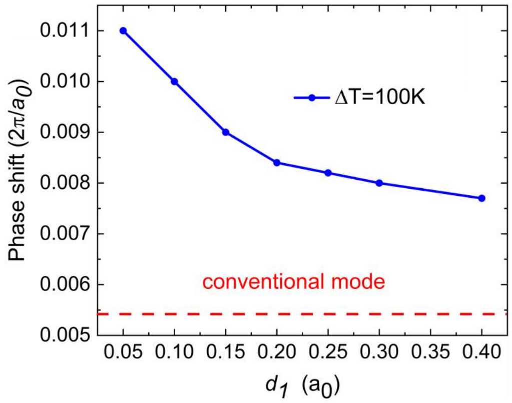

Fig. S1 Phase change of the topological edge mode with different side lengths of the small triangle (d_2) in the VPC.

Based on the differential value of the phase with temperature, $\Delta\phi_{topo} = -\frac{8\pi C_{s1}}{3a_0 n_{eff} C_{s2}} \frac{dn_{eff}(T)}{dT} l_{topo} \Delta T_1$. For the VPC structures based on a silicon layer with a stable thickness, we can obtain $\Delta\phi_{topo} \propto -\frac{C_{s1}}{C_{s2\,1}} = \frac{\beta_0 + \frac{1}{4}(\beta_1+\beta_2)}{\beta_0 - \frac{1}{2}(\beta_1+\beta_2)}$. Therefore, the phase shifting efficiency is related to the degree of symmetry breaking ($\beta_1 + \beta_2$). Furthermore, the degree of symmetry breaking can be presented by the side lengths of the small triangle (d_2) in the VPC. As shown in Fig. S1, the phase shift increases with decreasing d_2.

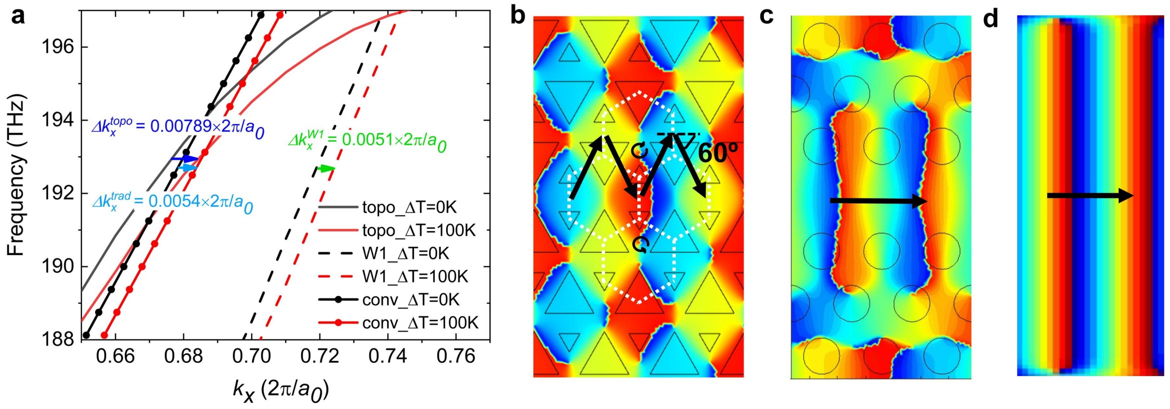

Fig. S2 a Temperature-dependent dispersion relations of modes in the conventional strip waveguide, W1 waveguide and topological edge 1. **b-d** The *Hz* phase of the mode in topological edge 1, the W1 waveguide and the conventional waveguide. The black arrow represents the direction of light propagation.

Furthermore, we compare nontopological photonic crystal (PhC) waveguides, such as the standard line-defect (W1) waveguide. The basic structure of W1 waveguide is shown in Ref. 8. Then, we simulate the energy band and obtain the temperature-dependent dispersion relations of valley edge state 1, the W1 waveguide and the conventional strip waveguide, respectively. The simulation results in Fig. S2a show that the phase change of the valley edge mode ($\Delta k_x^{topo} = 0.00789 \times 2\pi / a_0$) is 1.547 times that in the W1 waveguide ($\Delta k_x^{trad} = 0.0051 \times 2\pi / a_0$) with the same refractive index variation of silicon. In the W1 waveguide, the light

cannot propagate in the band gap of the photonic crystal; it is localized in the surroundings of the defect; i.e., the line defect acts as a waveguide. As shown in Fig. S2b-d, the length of the optical path of the topological mode in edge 1 is close to twofold that of the mode in the W1 waveguide and in the conventional strip waveguide, at λ = 1550 nm. Therefore, the higher phase shift efficiency of the topological mode can be attributed to the increased optical path length resulted from the phase vortices generated by the VPC structure.

Supplementary Note 2: Test of backscattering

To verify the topological protection of the valley edge state, we use a conventional DC to obtain the reflectance spectrum of the topological waveguide and line-defect PhC waveguide with sharp turns. An optical microscope photo and SEM image of the basic structures are shown in Fig. S3 a and b. The fabricated devices (enclosed by the dashed black box) are cascaded with the directional coupler for testing. We can obtain the transmission and reflectance spectra at Port 1 and Port 2, respectively. The spectra can be normalized to that of the 2×2 DC. The measured reflection of device 1 (conventional waveguide) at Port 2 is lower than -20 dB in the wavelength range of 1480 nm - 1580 nm, as illustrated in Fig. S3 c.

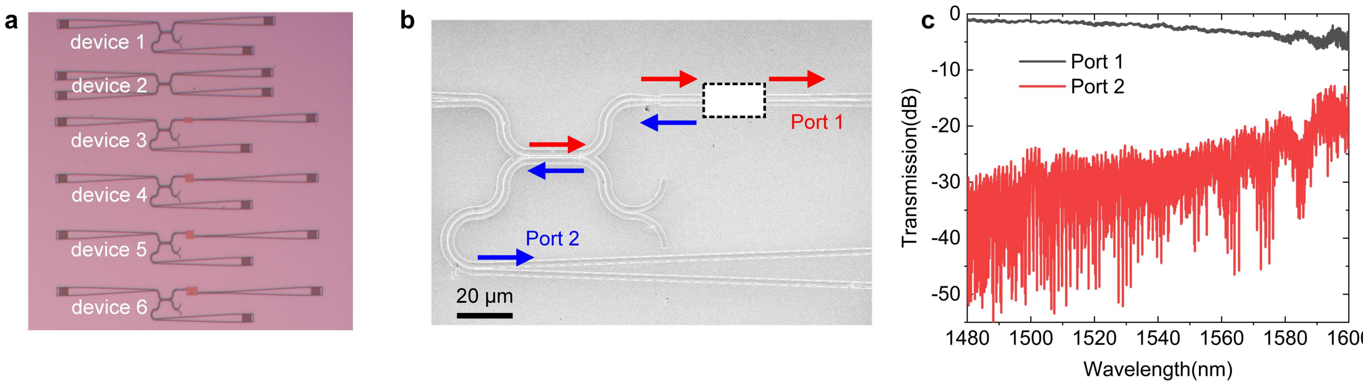

Fig. S3 Structures for testing the backscattering. **a** Optical microscope photo of the fabricated devices. **b** Scanning electron microscope (SEM) image of the conventional directional coupler (DC). The red arrows indicate the forward-propagating modes and blue arrows indicate backward-propagating modes before and after passing through the devices. The structures in the dashed black box of devices 3-6 are the straight topological waveguide, topological waveguide with a 60-degree turn, line-defect PhC waveguide and line-defect PhC waveguide with a 60-degree turn, respectively. The measured reflectance responses are normalized to those of device 2. **c** The transmission of device 1.

The reflectance spectra measured at Port 2 include the scattering at the end facet and the possible backscattering caused by the sharp turns. For topological devices 3 and 4, the transmission and reflection spectra are similar to those in Fig. S4 e and f, which suggests that the proposed edge state 1 is topologically protected against scattering during sharp turns. On the other hand, based on the transmission and reflection spectra in Fig. S4 g and h, we can deduce that the light is scattered during sharp turns in the line-defect PhC waveguide. The comparison confirms that only the topological mode can be protected against backscattering at sharp turns.

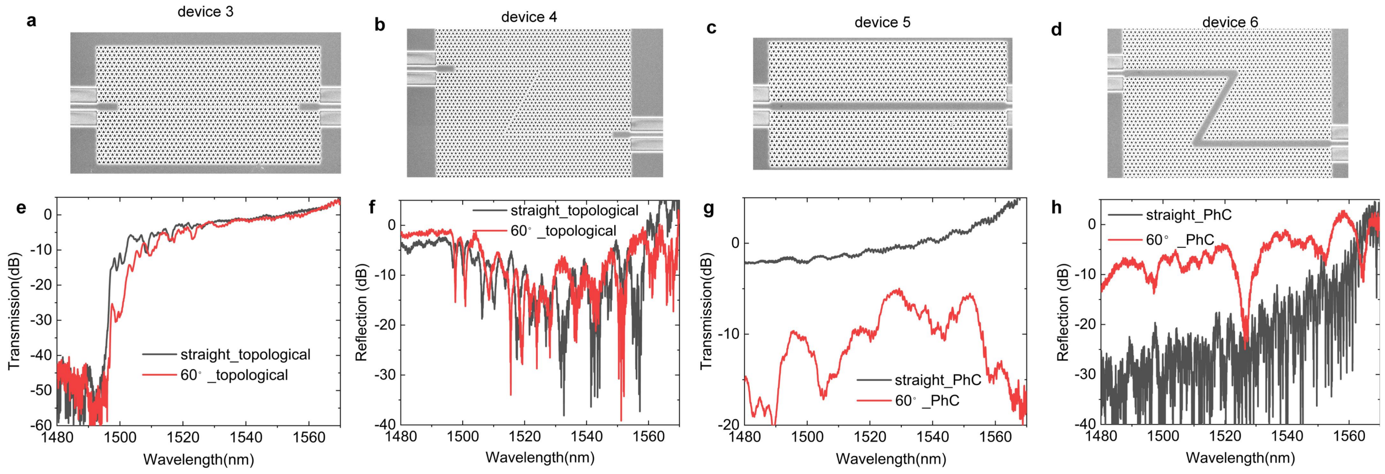

Fig. S4 Forward transport of light along sharp turns. **a-d** SEM images of device 3 (straight topological waveguide), device 4 (topological waveguide with a 60-degree turn), device 5 (line-defect PhC waveguide) and device 6 (line-defect PhC waveguide with a 60-degree turn). **e-h** Transmission and reflection of devices 3-6.

Supplementary Note 3: Comparison of phase shifting efficiency

We use conventional MZI structures to measure the π-phase shifting power of topological and conventional waveguides, as shown in Fig. S5 a and b. The MZI structures consist of two 50:50 power splitters and two different MZI arms. The width of the conventional waveguide is 790 nm. The whole device is covered with SiO_2. Titanium heaters 350-nm-thick and 2.4 μm wide are fabricated on top of the MZI arms. The heating length of the two kinds of phase shifters is 63.8 μm. By applying different voltages to the metal heaters, we can obtain the thermal tuning efficiency of the phase shifters based on the conventional and topological waveguides. The experimental results are shown in Fig. 2e of the main text.

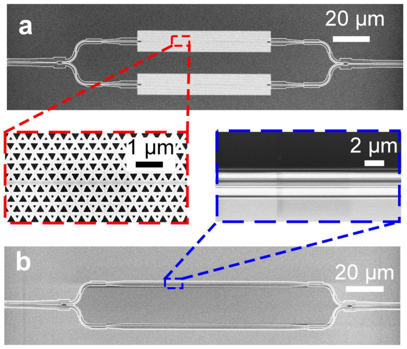

Fig. S5 Comparison of the phase shift efficiency. The conventional Mach–Zehnder interferometer (MZI) consists of two power splitters, **a** a topological phase shifter and **b** a conventional waveguide phase shifter. The insets enclosed by the red and blue dashed boxes are SEM images of topological and conventional waveguides, respectively.

Supplementary Note 4: Transmission loss of edge 1

In this section, we measure the transmission loss as a function of propagation length, which is shown in Fig. S6 a. Edge 1 is shown in Fig. 1 c in the main text. The loss is caused by out-of-plane scattering, as pointed out in Ref. 9. Fig. S6 b shows that the propagation loss along edge 1 is 0.047 dB/μm at λ= 1550 nm.

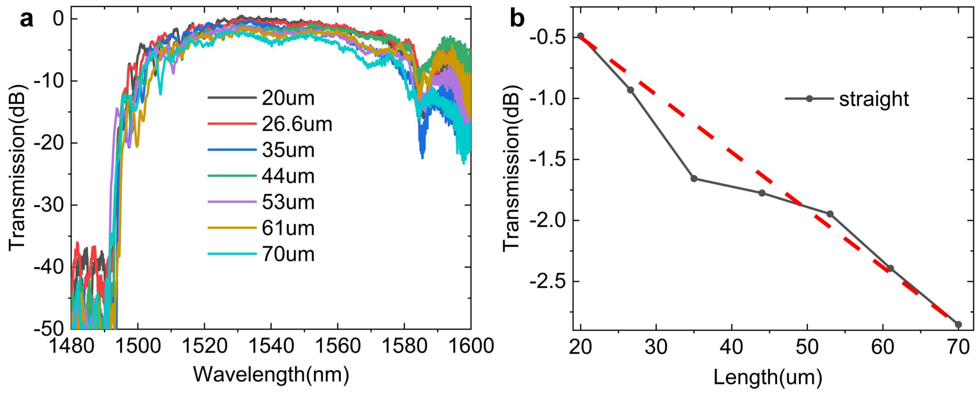

Fig. S6 Transmission loss of the fabricated topological waveguide. **a** Transmission of topological modes with different propagation lengths. **b** The length-dependent transmission at λ= 1550 nm.

Supplementary Note 5: Coupling theory of a topological power splitter

In this section, we discuss the interference at the coupling point of the topological power splitter, as shown in Fig. 3 c in the main text. The interference between the odd mode (edge 1) and even mode (edge 2) has been discussed in Ref. 9 and 10. The basic structures of edge 1 and edge 2 are shown in Fig. 1 c in the main text. Fig. S7 a shows that the light is output at Port 4 when the phases of light from input ports 1 and 2 are the same. On the other hand, if the difference in the phases between input ports 1 and 2 is π, the light is output from port 3, as shown in Fig. S7 b. Therefore, the interference phenomenon can be used to realize a 2× 2 TPS, as shown in Fig. 3 in the main text.

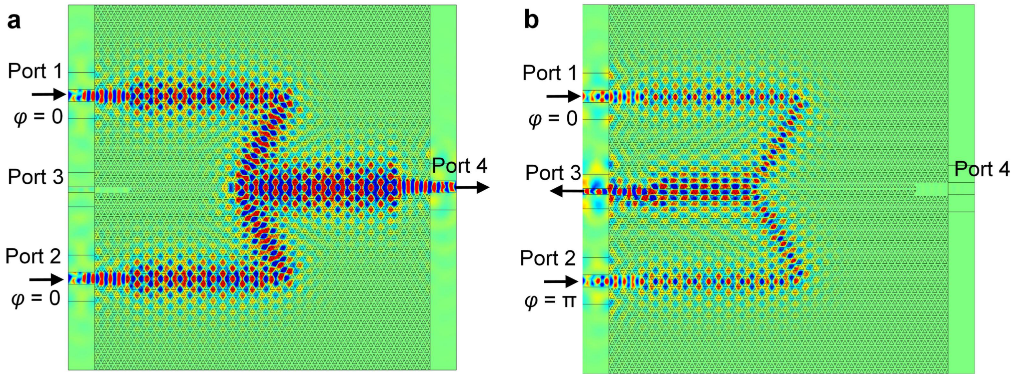

Fig. S7 Topological power splitter. **a, b** Simulated H_z field in the power splitter when the waves input from Port 1 and Port 2 are **a** in phase and **b** out of phase.

Supplementary Note 6: Experimental results of TMZI

In this section, we discuss the transmission of the thermo-optic (TO) tuned TMZI. The basic structure is shown in Fig. S8 a. When an extra phase change of π is added to one of the TMZI arms, interference occurs at the cross point, and light is forbidden from outputting from Port 1. By varying the heating length of the TMZI arm *l*, the π-phase shifting power changes, as shown in Fig. S8 b. Fig. S8 c shows that when *r* is 15 μm, the π-phase switching power is approximately 15 mW in the wavelength range from 1530 nm to 1580 nm. The working band is related to the dispersion relation of the topological waveguides. The phase change of the valley edge mode Δk_x^{topo} with a change in temperature $\Delta T = 100K$ can be obtained with the data indicated in Fig. 2 a of the main text. As shown in Fig. S8 d, the calculated difference in Δk_x^{topo} is $(0.006 \sim 0.01) \times 2\pi / a_0$ at frequencies of 189.4 THz to 195 THz.

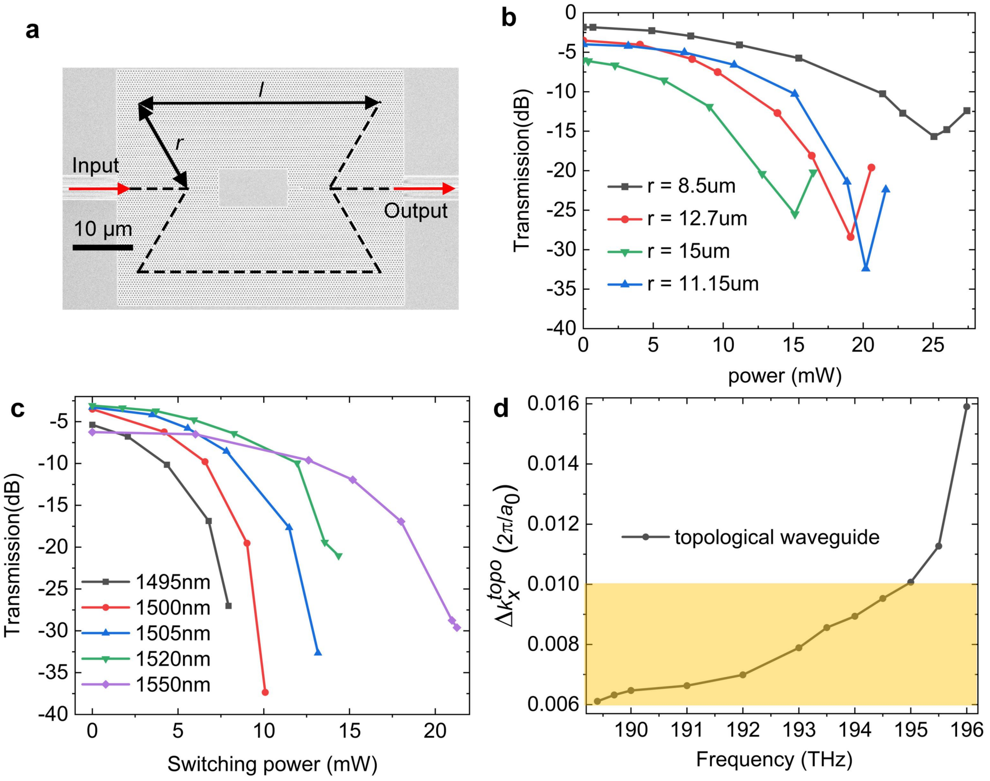

Fig. S8 Measurement of the fabricated TMZI. **a** SEM image of TMZI. The heating length of the topological waveguide is l=2r+24.5 μm. **b** Switching characteristics of the TMZI with different r. **c** Switching characteristics of the TMZI at different wavelengths when r = 15 μm. **d** Phase change of the topological edge mode at different frequencies with the change in temperature $\Delta T = 100K$.

Supplementary Note 7: Design of the crossing structure

The structure of the optimized crossing is shown in Fig. S9 a. Unlike the conventional method of optimizing the boundary of the structure using the particle swarm optimization algorithm, we perform a Fourier expansion on the boundary of one-eighth of the crossing and optimize the Fourier coefficients. In practice, the length of a one-eighth crossing is 2 μm, and the coefficients of the

first six Fourier series whose periods are larger than the Bragg condition (~ 0.33 μm) are taken for optimization. As plotted in Fig. S9 b, the measured insertion loss and crosstalk of the optimized crossing remain below 0.1 dB and −22 dB in the wavelength range of 1480–1600 nm.

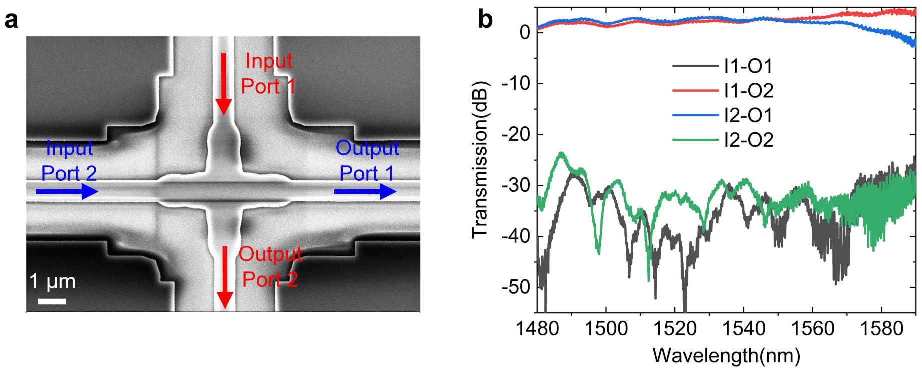

Fig. S9 Crossing structure. **a** SEM image of the crossing structure. **b** Experimental insertion loss and cross-talk spectra of the crossing structure.

Supplementary Note 8: Fabrication process and optical characterization setup

This section shows the fabrication process and the experimental setup for our devices. We fabricated the proposed topological devices on an SOI wafer (220-nm thick silicon on 3000-nm thick silica)[11]. E-beam lithography (EBL) (Vistec EBPG 5200$^+$) and an inductively coupled plasma (ICP) etching process (SPTS DRIE-I) were used to pattern and etch grating couplers, silicon waveguides, VPC structures and heaters. Fig. S10 a shows the fabrication process of the topological devices. The structures of the waveguide and the VPC were first defined on an e-beam resist using EBL. The pattern was then transferred to the top silicon layer, and the depth of dry etching was ~ 220 nm. Subsequently, EBL and ICP dry etching were used to define the grating coupler on the silicon layer with an etching depth of ~ 70 nm. The period and duty cycle of the grating coupler were 630 nm and 50%, respectively. Then, the whole device was covered with 1-μm-thick SiO_2. A 350-nm-thick platinum heater was sputtered above the SiO_2. Two pads were coated with 350-nm-thick gold through evaporation and connected to the heaters as electrodes.

The experimental setup for the device characterization is shown in Fig. S10 b. We used a tuneable continuous wave (CW) laser source (Santec TSL770) covering a 140-nm wavelength range from 1480 to 1620 nm to characterize the performances of the proposed devices. A polarization controller was utilized to adjust the polarization state of the incident light in the optical fibre. To couple light into and out of the chip, a vertical coupling configuration was adopted. The measured coupling losses of the TE-polarized grating coupler were 8.1 dB/facet, with a 3-dB bandwidth of 55 nm. Then, an optical power metre was used to record the power of the output signal. A voltage-current source-metre (Keithley 2400) was used to supply the thermal tuning power for the TPS. We varied the voltage applied to the metal heater to achieve π-phase shifting.

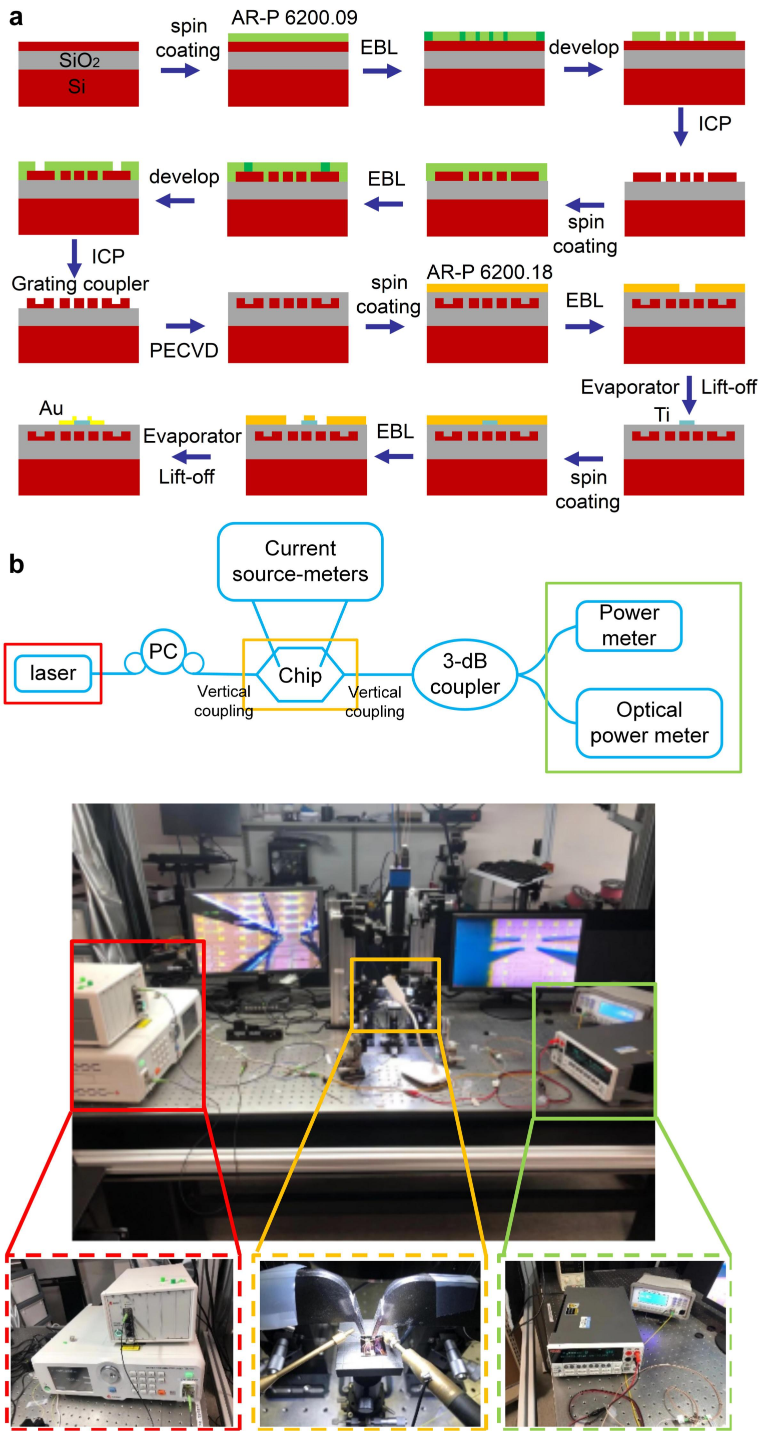

Fig. S10 Fabrication process and measurement setup. **a** Device fabrication process. EBL: e-beam lithography. ICP: inductively coupled plasma. **b** Experimental setup for topological device characterization.

Reference:

1. Ma, T. & Shvets, G., All-Si valley-Hall photonic topological insulator, *New J. Phys.* **18**, 025012 (2016).
2. Chen, X. D., Zhao, F. L. Chen, M. & Dong, J. W., Valley-contrasting physics in all-dielectric photonic crystals: Orbital angular momentum and topological propagation, *Phys. Rev. B* **96**, 020202 (2017).
3. Shalaev, M. I., Walasik, W., Tsukernik, A., Xu, Y. & Litchinitser, N. M. Robust topologically protected transport in photonic

crystals at telecommunication wavelengths. *Nat. Nanotechnol.* **14**, 98-98 (2019).
4. Barik, S., Miyake, H., DeGottardi, W., Waks E. & Hafezi, M., Two-dimensionally confined topological edge states in photonic crystals, *New J Phys.* **18**, 113013 (2016).
5. Smirnova, D. et al. Room-temperature lasing from nanophotonic topological cavities. *Light Sci. Appl* **9**, 127 (2020).
6. Chen, Y., et al. Topologically protected valley-dependent quantum photonic circuits. *Phys. Rev. Lett.* **126**, 230503 (2021).
7. He, X. T. et al. A silicon-on-insulator slab for topological valley transport. *Nat. Commun.* **10**, 872 (2019).
8. Hauff, N. V., Jeannic, H. L. & Lodahl, P., Chiral quantum optics in broken-symmetry and topological photonic crystal waveguides. *Phys. Rev. Res.* **4**, 023082 (2022).
9. Wang, H. W. et al. Asymmetric topological valley edge states on silicon-on-insulator platform, *Laser Photonics Rev.* **16**, 2100631, 1-8 (2022).
10. He, L., Zhang, W. X. & Zhang, X. D., Topological all-optical logic gates based on two-dimensional photonic crystals, *Opt. Express* **27**, 25841–25859 (2019).
11. Su, Y. K., Zhang, Y., Qiu, C. Y., Guo, X. H. & Sun, L. Silicon photonic platform for passive waveguide devices: materials, fabrication, and applications. *Adv. Mater. Technol.* **5**, 1901153, 1-19 (2020).
